# Black Hairy Tongue Associated With Linezolid Treatment for Olecranon Bursitis Due to Streptococcus salivarius and Streptococcus mitis

**DOI:** 10.7759/cureus.87745

**Published:** 2025-07-11

**Authors:** Chandler Q Kotseos, Michael K Seifert

**Affiliations:** 1 UNC Orthopaedics, University of North Carolina at Chapel Hill School of Medicine, Chapel Hill, USA

**Keywords:** adverse drug reaction, black hairy tongue, elbow pain, linezolid, olecranon bursitis, streptococcus mitis, streptococcus salivarius

## Abstract

A 63-year-old female presented with acute, atraumatic posterior elbow pain. She was found to have infectious olecranon bursitis with resistant bacteria. Her treatment was complicated by a penicillin allergy, necessitating the use of linezolid, which was further complicated by the rare black hairy tongue (BHT) side effect of linezolid. Despite the visible discoloration and associated pain from BHT, the patient continued linezolid to treat her bursal infection. She recovered from the bursitis and linezolid side effects after completion, supporting that immediate withdrawal is not necessary for the resolution of BHT but should be part of shared decision-making to avoid early discontinuation of the antimicrobial.

## Introduction

Linezolid is an oxazolidinone antibiotic that is effective against many different Gram-positive bacteria, including methicillin-resistant Staphylococcus aureus (MRSA) and vancomycin-resistant Enterococci (VRE) [1,2]. The mechanism of action involves binding to the 50S subunit on bacterial 23S ribosomal RNA to prevent translation and thus protein synthesis. By specifically targeting bacterial proteins, linezolid is associated with few common side effects, which include nausea, vomiting, diarrhea, headache, taste changes, and rash. With prolonged use, bone marrow suppression, peripheral neuropathy, and rarer complications, such as black hairy tongue (BHT), seen in 0.2% of phase III clinical trials, have been reported [2].

BHT is a clinical diagnosis based on hypertrophy and discoloration of the filiform papillae on the dorsal surface of the tongue, which has been described as black, brown, green, and blue [1,2]. Although the exact mechanism of BHT is unclear, it is proposed to be caused by defective desquamation, resulting in thickened papillae that collect debris, bacteria, and fungus, leading to the color change. When caused by linezolid, the condition consistently resolves after stopping the antibiotic. This case provides a scenario where the benefits of linezolid therapy in treating resistant olecranon bursitis outweighed its side effects.

## Case presentation

A 63-year-old female was seen in an outpatient clinic with left elbow pain. She had woken that morning with sudden-onset posterior elbow pain without known trauma. She noted warmth in the area but denied fevers, chills, and malaise. She occasionally gardened and walked regularly, but did not perform regular repetitive upper extremity exercises. She had a medical history significant for atrial fibrillation, hypertension, idiopathic small fiber neuropathy, idiopathic thrombocytopenic purpura (ITP), and knee osteoarthritis. She had no surgical history. She has an anaphylactic allergy to penicillin.

**Figure 1:**
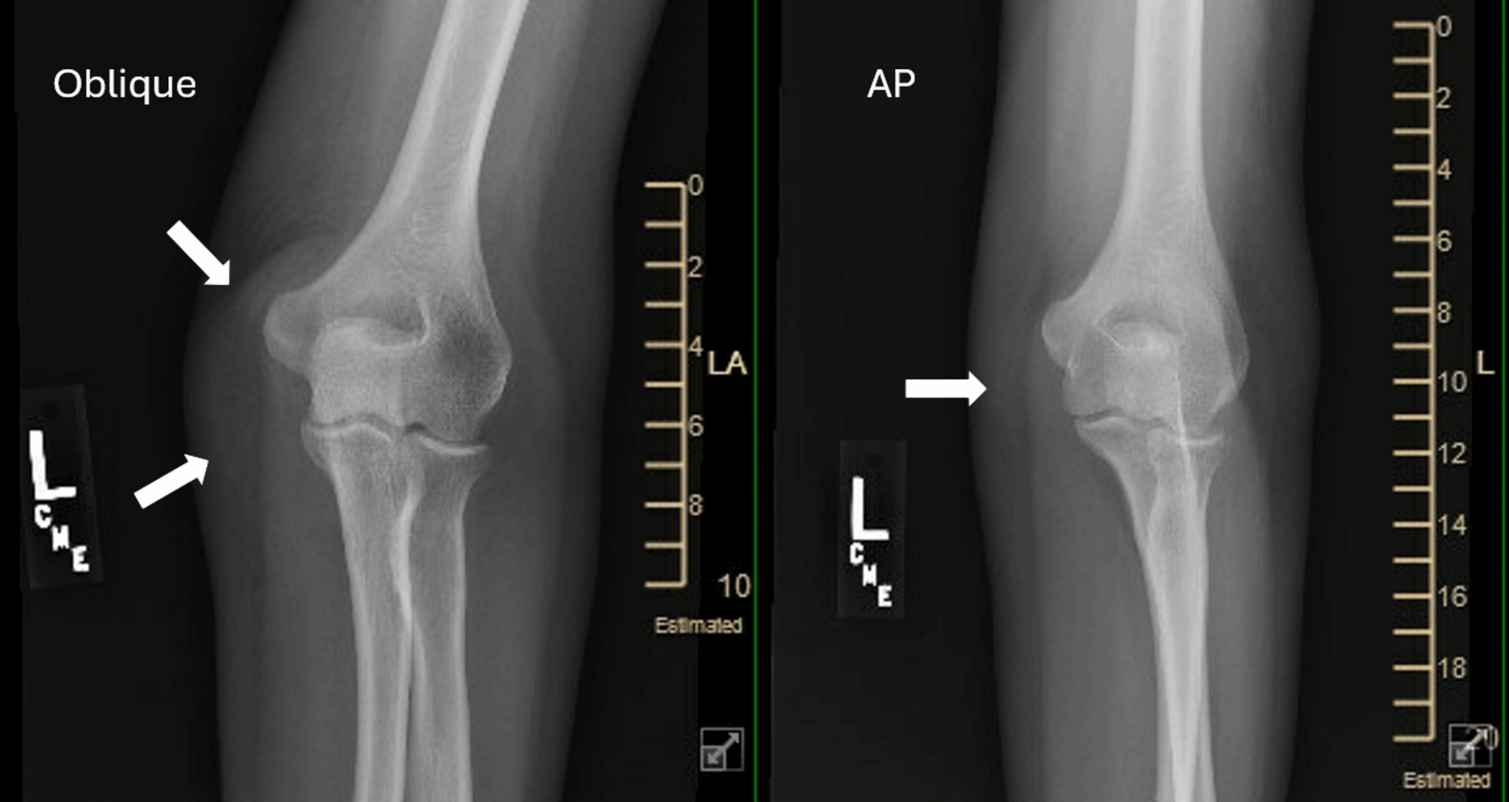
Oblique and AP films of the elbow taken during the initial evaluation of left elbow pain AP: anteroposterior

**Figure 2:**
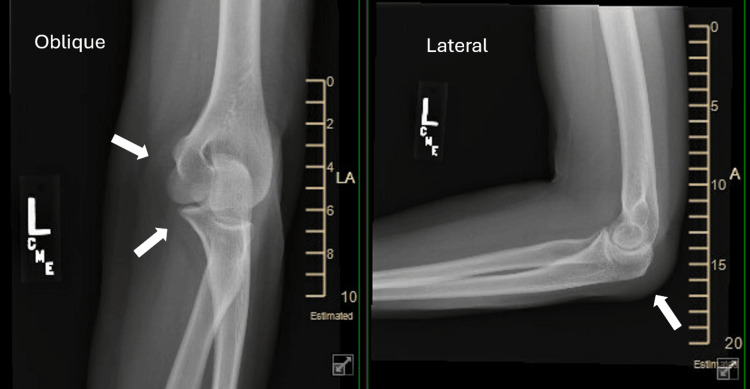
Oblique and lateral films of the elbow taken during initial evaluation of left elbow pain

On physical exam, there was edema over the olecranon with surrounding erythema, palpable warmth, and severe tenderness. No skin opening or wound was noted. She had full extension and flexion, with pain at the extremes. The X-ray showed no fracture with soft tissue swelling posterior to the olecranon, suggesting bursitis (Figure 1 and Figure 2). Aspiration of the bursa produced 4 mL of straw-colored fluid that was sent for culture and sensitivity.

Bursal fluid analysis showed a high neutrophil count and high overall cell count (Table 1), and antibiotic treatment was started prior to the finalization of culture results. A pharmacist was consulted about treatment that would not interact with flecainide and was not penicillin, and sulfamethoxazole-trimethoprim (TMP-SMX) 400 mg - 80 mg, 2 pills by mouth, twice daily, was prescribed, which did not change the appearance of her elbow after five days. Culture results later indicated *Streptococcus salivarius* and *Streptococcus mitis*, and *Streptococcus mitis* was resistant to clindamycin. An infectious disease (ID) physician was consulted, commenting that TMP-SMX is generally not an acceptable treatment for streptococcal infection, and suggesting vancomycin as an appropriate inpatient option. However, this patient was stable and did not require admission, so the consultant ultimately suggested linezolid 600 mg by mouth, twice daily for 10 days as an acceptable alternative with serial aspirations and close clinical monitoring. Our consultant did not consider BHT, presumably due to its rarity, but did weigh the possibility of linezolid decreasing platelet counts given the patient’s history of ITP. However, she has had stable platelet counts for over a year. The consultant suggested that a short course of linezolid would likely be tolerated well.

**Table 1:** Bursal fluid analysis results

Parameter	Result	Reference Range
Nucleated cells	27,227 cells/μL	< 500 cells/μL
Neutrophil percentage	80%	< 25%
Lymphocyte percentage	4%	10–30%
Monocyte/macrophage percentage	14%	Variable
RBC count	7,439 cells/μL	< 2,000 cells/μL

**Figure 3:**
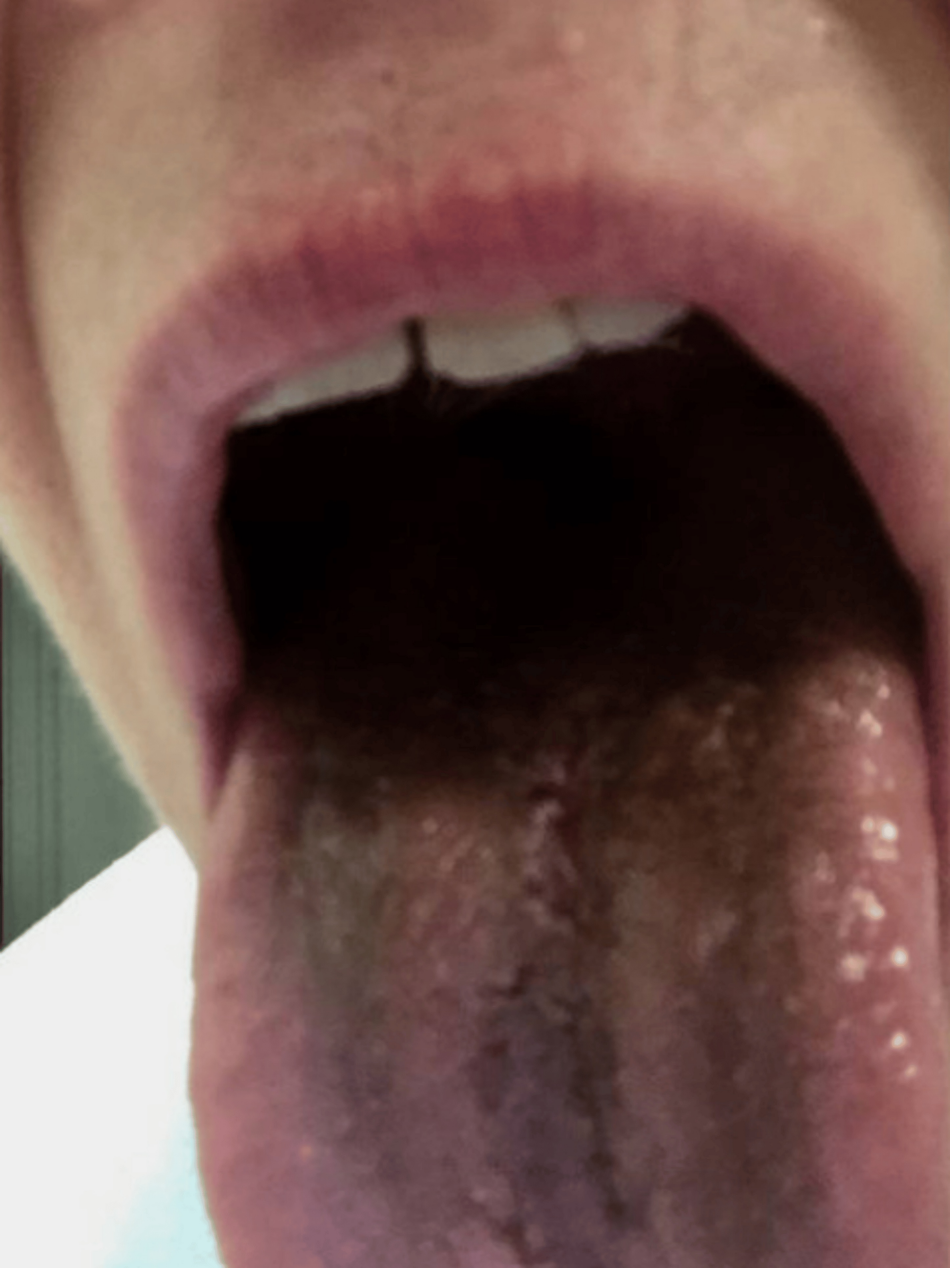
Dark discoloration of the tongue after one week of linezolid therapy

The patient returned to the clinic four days after starting linezolid, with significantly improved skin erythema around the olecranon but recurrent olecranon bursal swelling. A repeat aspiration of the bursa obtained 2 cc of dark serous fluid. She was encouraged to finish her antibiotic course and woke up three days later with a black tongue (Figure 3), seven days after starting linezolid. She reported painful cracks and bumps along her tongue and stated that her elbow refilled with fluid; she later reported stomach pain and yeast vaginitis as side effects from linezolid as well. She returned to the clinic the next day, where aspiration yielded 2 cc of sanguinous fluid, and finishing linezolid was recommended for the last day and a half of the prescription.

An adverse drug reaction (ADR) analysis was performed using Naranjo’s algorithm to assess the likelihood of linezolid-induced BHT, which produced a score of 6, indicating probable ADR [3]. No biopsy of the tongue was taken based on contact with a pharmacist who found case reports of BHT associated with linezolid, suggesting that the discoloration resolves after completion of the antibiotic course. At a later visit for a separate issue, the patient reported that the BHT and linezolid’s other side effects of stomach pain and yeast vaginitis resolved immediately after antibiotic completion.

## Discussion

Linezolid-induced black hairy tongue (BHT) is a rare, benign adverse reaction with aesthetic implications for patients that can cause premature discontinuation of the antibiotic and necessitates effective counseling. While the safety profile of linezolid is well-established, some studies and case reports suggest that the rate of BHT in broader comparator-controlled studies is closer to 1% [4,5], but the precise incidence remains unclear due to limited primary source data. The average time after starting linezolid therapy until the diagnosis of BHT is two weeks, but it can be seen earlier, like in this case (i.e., seven days), and in as little as two days. The side effect typically resolves seven days after stopping the antibiotic [1]. Although BHT is usually asymptomatic, it can be accompanied by a tickling or burning sensation, halitosis, dysgeusia, and nausea, further contributing to discontinuation [1]. Nevertheless, several cases have documented full completion of the course of antibiotics after BHT diagnosis based on provider recommendation and a shared decision with the patient [1,2,6]. This case demonstrates an example of such shared decision-making, specifically regarding the risk-benefit assessment of continuing linezolid despite BHT with tongue and stomach pain as side effects.

The nature of this patient’s infection and empirical improvement after starting linezolid were a key factor in her decision to continue the medication. *Staphylococcus aureus* is by far the most common causative organism of olecranon bursitis, and even though *Streptococcus* is the second most reported genus, the *Streptococcus* *salivarius* and *Streptococcus* *mitis* species specifically are scarce in existing literature [7]. This presented a unique challenge in treatment, which was amplified by a severe allergy to penicillin, the ineffectiveness of TMP-SMX, and *Streptococcus* *mitis* resistance to clindamycin. The patient was aware of these difficulties in treatment and the peculiarity of the laboratory results, which likely contributed to her agreement to complete the linezolid course after notable symptomatic improvement of her bursitis. In addition, some alternative options to manage BHT are possible causative agents, such as oxygenating mouth rinses, which further complicates treatment [8]. Investigators have suggested brushing the dorsum of the tongue twice daily with 3% hydrogen peroxide or baking soda, and others have recommended topical triamcinolone acetonide or antifungal agents, but there is sparse evidence for an effective cure besides the discontinuation of the offending agent. Regardless, patient counseling should include these alternatives to handle such side effects without treatment interruption despite the absence of a clear consensus.

This patient did not have any significant risk factors documented for linezolid-induced BHT, including tobacco use, cancer, acquired immune syndrome, or concomitant antibiotic use [2], and she understood the self-limiting nature of BHT after reassurance from the pharmacist and physician. Furthermore, it is important to highlight other daily risk factors, like black tea or coffee consumption and poor oral hygiene, so that patients can avoid an aggravation of the condition or reduce the risk of recurrence. These controllable predisposing factors, like tobacco use and dark beverages, should be eliminated while maintaining proper brushing and flossing habits to minimize the exacerbation of BHT. Moreover, this patient’s full recovery after linezolid completion shows that immediate withdrawal of the offending agent may not be essential for the management of BHT, contrary to the approach offered in prior reports, such as Balaji et al. [9], and the adverse effect may be outweighed by the benefits of treating the original infection.

The mechanism of linezolid-induced BHT is poorly understood, and its occurrence is infrequently reported, requiring more documentation on associated conditions and pathophysiologic investigation. However, it is still worth counseling about this and other rarer side effects so that they are prepared for the possibility. While the management is generally supportive, with an emphasis on proper dental hygiene, persistent cases have occurred and were resolved by brushing with baking soda or hydrogen peroxide, and topical agents, such as urea, trichloroacetic acid, thymol, and B vitamins, have been reported as effective [10]. These additional conservative treatment options must be communicated to patients and play into their decision-making process as more evidence is gathered for guidelines in the management of linezolid-induced BHT. Physicians should simultaneously be sensitive to the aesthetic concerns of patients and offer alternative management options to avoid the premature discontinuation of linezolid.

## Conclusions

This case highlights the importance of clinical collaboration and shared decision-making with the patient when faced with uncommon drug reactions. While BHT is generally benign, its aesthetic impact can influence adherence and complicate treatment decisions. In this case, the unusual presentation of olecranon bursitis caused by non-traditional streptococcal species further added to the complexity of the management plan. Through consultation and counseling, the patient elected to complete treatment with linezolid despite the discomfort, underscoring the value of multidisciplinary patient education to optimize outcomes in rare clinical scenarios. This case also identifies an opportunity for proactive counseling about adverse drug reactions to avoid unnecessary discontinuation. Given the unclear mechanism underlying BHT and the lack of formal management guidelines, further research and case reports are needed to inform best practices.

## References

[1] Khasawneh FA, Moti DF, Zorek JA (2013). Linezolid-induced black hairy tongue: a case report. J Med Case Rep.

[2] Sethi Y, Padda I, Fulton M, Kaiwan O, Chopra H, Bin Emran T (2023). Linezolid-induced black hairy tongue in a patient treated for idiopathic granulomatous mastitis: a case report. Ann Med Surg (Lond).

[3] Naranjo CA, Busto U, Sellers EM (1981). A method for estimating the probability of adverse drug reactions. Clin Pharmacol Ther.

[4] Pedada SP, Allamsetty J, Bammidi T, Viriti US (2022). Case study on linezolid induced black hairy tongue. Asian Jour Hosp Phar.

[5] Aijazi I, Abdulla FM (2014). Linezolid induced black hairy tongue: a rare side effect. J Ayub Med Coll Abbottabad.

[6] Lee J, Chung HS, Roh J, Oh Y, Mok J (2021). Linezolid-induced black hairy tongue in a patient with multidrug-resistant tuberculosis: a case report. Sci Prog.

[7] Reilly D, Kamineni S (2016). Olecranon bursitis. J Shoulder Elbow Surg.

[8] Lawoyin D, Brown RS (2008). Drug-induced black hairy tongue: diagnosis and management challenges. Dent Today.

[9] Balaji G, Maharani B, Ravichandran V, Parthasarathi T (2014). Linezolid induced black hairy tongue. Indian J Pharmacol.

[10] Mishra GP (2023). Linezolid-associated black hairy tongue: a rare adverse drug reaction. J Infectiology.

